# Impact of commissural calcification on clinical outcome of percutaneous balloon mitral valvuloplasty; a retrospective cohort study of 876 patients

**DOI:** 10.1186/s12872-024-03932-w

**Published:** 2024-06-18

**Authors:** Saman Rostambeigi, Homa Mazaherinia, Negin Mahmoudi Hamidabad, Anita M. Kelsey, Azin Alizadehasl, Seyed Taha Hosseini Harandi, Khashayar Farnoud, Parsa Panahi, Ata Firouzi, Anita Sadeghpour

**Affiliations:** 1 Rajaie Cardiovascular, Medical, and Research Center, Iran University of Medical Sciences, Cardiologist, Tehran, Iran; 2 School of Medicine, Department of Internal Medicine, Rasoul-E-Akram Hospital, Iran University of Medical Sciences, Tehran, Iran; 3 Division of Cardiology and Duke Heart Center, Duke University Medical Center, Durham, NC England; 4 Cardio-Oncology Department and Research Center, Rajaie Cardiovascular Medical and Research Center, Tehran, Iran; 5 Student Research Committee, School of Medicine, Iran University of Medical Sciences, Tehran, Iran; 6 MedStar Cardiovascular Core Lab, MedStar Health Research Institute, Georgetown University, Washington, DC US

**Keywords:** Percutaneous balloon mitral valvuloplasty (PBMV), Commissure calcification, Mitral valve, Mitral Stenosis, Rheumatic Valve Disease

## Abstract

**Background:**

Percutaneous balloon mitral valvuloplasty (PBMV) is the ACC/AHA class I recommendation for treating symptomatic rheumatic mitral stenosis with suitable valve morphology, less than moderate MR and absence of left atrium clot. The mitral valve restenosis and significant mitral regurgitation (MR) are known adverse outcomes of PBMV. This study aimed to evaluate the outcomes of PBMV in patients with severe mitral stenosis and the effect of Commissural Calcification (CC) on the outcomes.

**Methods:**

In this single-center retrospective cohort study, 876 patients who underwent PBMV were categorized into three groups based on their Wilkins score (Group I: score ≤ 8, Group II: score 9–10, and Group III: score 11–12). Patients were evaluated before, early after PBMV and at 6- and 24-month follow-ups. Main clinical outcomes were defined as significant restenosis and or symptomatic significant MR (moderate to severe and severe MR) or candidate for mitral valve replacement (MVR). The outcomes were compared between patients with and without CC.

**Results:**

A total of 876 patients with mean age 46.4 ± 12.3 years (81.0% females) were categorized based on Wilkins score. 333 (38.0%) were in Group I, 501 (57.2%) were in Group II, and 42 (4.8%) were in Group III. CC was present in 175 (20.0%) of the patients, among whom 95 (54.3%) had calcification of the anterolateral commissure, 64 (36.6%) had calcification of the posteromedial commissure, and in 16 (9.1%) patients both commissures were calcified. There was a significant difference in Wilkins score between patients with and without CC (*P* < 0.001). CC was associated with higher odds of significant symptomatic MR at early and mid-term follow up (OR: 1.69, 95%CI 1.19–2.41, *P* = 0.003; and OR: 3.90, 95%CI 2.61–5.83, *P* < 0.001, respectively), but not with restenosis (*P* = 0.128). Wilkins Groups II and III did not show higher odds of significant symptomatic MR compared to Group I at early (II: *P* = 0.784; III: *P* = 0.098) and mid-term follow up (II: *P* = 0.216; III: *P* = 0.227). Patients in Wilkins Group II had higher odds of restenosis compared to Group I (OR: 2.96,95%CI: 1.35–6.27, *P* = 0.007).

**Conclusion:**

Commissural calcification (CC) is an independent predictor of the significant symptomatic MR (an important determinant of adverse outcome) following PBMV in the early and mid-term follow-up. Mitral valve restenosis occurs more in patients with higher Wilkins score compared to group I with score ≤ 8. Combined Wilkins score and CC should be considered for patient suitability for PBMV.

## Introduction

While rheumatic mitral stenosis (MS) is not a significant burden of cardiovascular disease in developed countries, it has remained one of the most common cause of heart valve disease in developing countries [1]. Untreated mitral stenosis is associated with poor prognosis, and may lead to complications such as pulmonary hypertension, atrial fibrillation and heart failure [2].

Percutaneous balloon mitral valvuloplasty (PBMV) is the ACC/AHA class I recommendation for the treatment of symptomatic rheumatic mitral stenosis with suitable valve morphology, less than moderate MR, and absence of left atrium clot [3]. A successful PBMV will result in an increase in mitral valve area (MVA) without significant MR [4–7]. However, complications associated with PBMV, including significant MR, minimal improvement in clinical status or MVA, underscore the need for a nuanced preprocedural evaluation and correlation with the outcomes [4, 5]. MR is the one of the most frequent complications of PBMV [6]. While the severity of MR after PBMV is often mild, more severe MR occurs in about 20% of the patients [7, 8]. Other complications of PBMV, such as embolic stroke, cardiac tamponade, and cardiac perforation, are rare. The role of echocardiographic characteristics of the mitral valve in predicting the severity of MR after PMV is controversial [9–12]. However, structural characteristics of the mitral valve are the most important factors for predicting the outcomes after PMV. The Wilkins score system is widely used to assess the suitability of the mitral valve leaflets for PBMV based on their morphological features including leaflet mobility, subvalvular thickening, leaflet thickening, and leaflet calcification [13, 14]. This scoring system is widely used to identify the patients who will benefit from PBMV as lower Wilkins scores are associated with a better-than-average prognosis after PBMV [8]. The main outcomes of PBMV include mitral valve regurgitation and mitral valve restenosis [9]. While the structure of the valvular commissures is not included in the Wilkins scoring system [15, 16], current studies have shown its association with the occurrence and severity of post-procedural MR [17, 18] and restenosis [10]. In vivo and in vitro studies have confirmed that commissural splitting is the dominant mechanism by which mitral valve area (MVA) is increased during balloon dilatation [11, 12]. This underscores the importance of a more thorough assessment of CC as a predictive factor for post-procedural complications. In this study, we aimed to evaluate MV CC impact on the short-term and mid-term outcomes after PBMV and to compare its predictive value with the conventional Wilkins score system. We hypothesized that the presence of CC would significantly predict the incidence of MR at short-term and mid-term follow-ups and might predict the occurrence of restenosis after PMV.

## Methods

### Study design and population

In this retrospective Cohort single-center study, 995 symptomatic moderate to severe and severe mitral stenosis (MVA ≤ 1.5 cm2) patients who underwent PBMV between 2009 and 2019 at Rajaie Cardiovascular, medical, and Research Center in Tehran, Iran, were enrolled. Exclusion criteria were MR (Moderate or severe), left atrial/ left atrial appendage thrombosis, Wilkins score > 12, concomitant coronary artery disease, and a history of previous valvular surgery. Patients who had major complications including tamponade, valve rupture, severe MR within the first 24 h after PBMV, and patients who were lost to follow-up (during the first 6 months after successful procedure) were excluded. All remaining patients were included in data analysis.

### Clinical assessment and follow-up

All patients were evaluated using transthoracic echocardiography (TTE) at the echocardiography unit of Rajaie cardiovascular, medical, and research center before and 24 h after PBMV to confirm the success of the procedure. All measurements were performed by expert cardiac sonographer and reviewed by a level III echocardiologist based on the recommendations of the American Society of Echocardiography guidelines [15]. MVA by planimetry and pressure half-time was measured in all patients.

All patients underwent PBMV using a self-positioning single balloon (Inoue balloon) and a step-wise dilation strategy based on current guidelines [7]. The upper limit of the balloon diameter was decided according to the patient’s height. Successful PMV was defined as post-PBMV > 1.5 cm^2^ or at least a 25% increase in the valve area with less than one grade increase in MR and without any major complications such as tamponade or cardiac perforation. MR severity was assessed by echocardiography using integration of multiple echocardiographic parameters and scored as absent or trivial, mild, moderate, moderate to severe, and severe [13, 14]. An ultrasound evaluation of the mitral valve structural features and subvalvular apparatus was performed for each patient before the procedure, supervised and interpreted by a level III echocardiographer, Wilkins echocardiographic scoring system was used to evaluate mitral valve structure. This system consists of 4 parameters: (a) leaflet thickening, (b) leaflet mobility, (c) leaflet calcification, and (d) subvalvular thickness. Each component is assigned a score from 0 to 4, and summing the individual scores results in a total mitral valve Wilkins score that ranges from 0 to 16. Patients were divided into three subgroups based on the Wilkins score. Group I consisted of patients with a Wilkins score ≤ 8, Group II included patients with a Wilkins score of 9–10, and Group III consisted of patients with a Wilkins score of > 10. Also, we have grouped the patients into two subgroups based on the presence or absence of CC. CC was determined visually when there was a bright echo density (higher signal density compared to the adjacent structure) on mitral valve commissures by 2D TTE. The presence of shadow behind the calcification was a confirmatory sign of calcification but not applicable for all subjects [11, 15].

### Study outcomes

The presence of symptomatic significant MR (moderate to severe or severe) that patient was candidate for surgical Mitral valve replacement (MVR) and/or mitral valve restenosis (defined as a 50% reduction in MVA after successful PBMV) served as the study outcomes. Symptomatic MR was defined as dyspnea New York Heart Association (NYHA) class II or greater [16]. Patients were followed with TTE and screened for MR severity at 6 and 24 months and for restenosis at 24 months. Incidence of MR and restenosis were compared based on the presence or absence of CC and based on the Wilkins score categories to identify the association between CC and Wilkins score with short-term and mid-term outcomes after PMV.

### Statistical analysis

Normally distributed continuous variables are described with mean ± SD and were compared with student T-Test, while non-normally distributed continuous variables are described with median [IQR] and were compared using the Mann-Whitney U test. Categorical variables are described as number (percent) and were compared suing the Chi-square or Fisher’s exact tests. The Wilcoxon test was used to compare echocardiologic parameters over time. Pearson test was used to find the correlations between the variables.

Univariable binary logistic regression analysis was used to explore the association between predictors (CC and Wilkins score categories) and study outcomes. Multivariable binary logistic regression models were fitted to assess the association between predictors and study outcomes after adjusting for covariates. For the multivariable models, X^2^, degrees of freedom, and P-values were reported, and odds ratios, 95% confidence intervals and P-values were reported for each predictor. Nagelkerke’s R^^^2 was used to quantify the contribution of predictors to the outcome. The Hosmer and Lemeshow test was used to assess goodness of fit and Akaike information criterion was applied to avoid overfitting.

Statistical analysis was performed using SPSS version 28 and R version 4.3.2. A P-value less than 0.05 was considered significant.

## Results

### Baseline characteristics

**Fig. 1:**
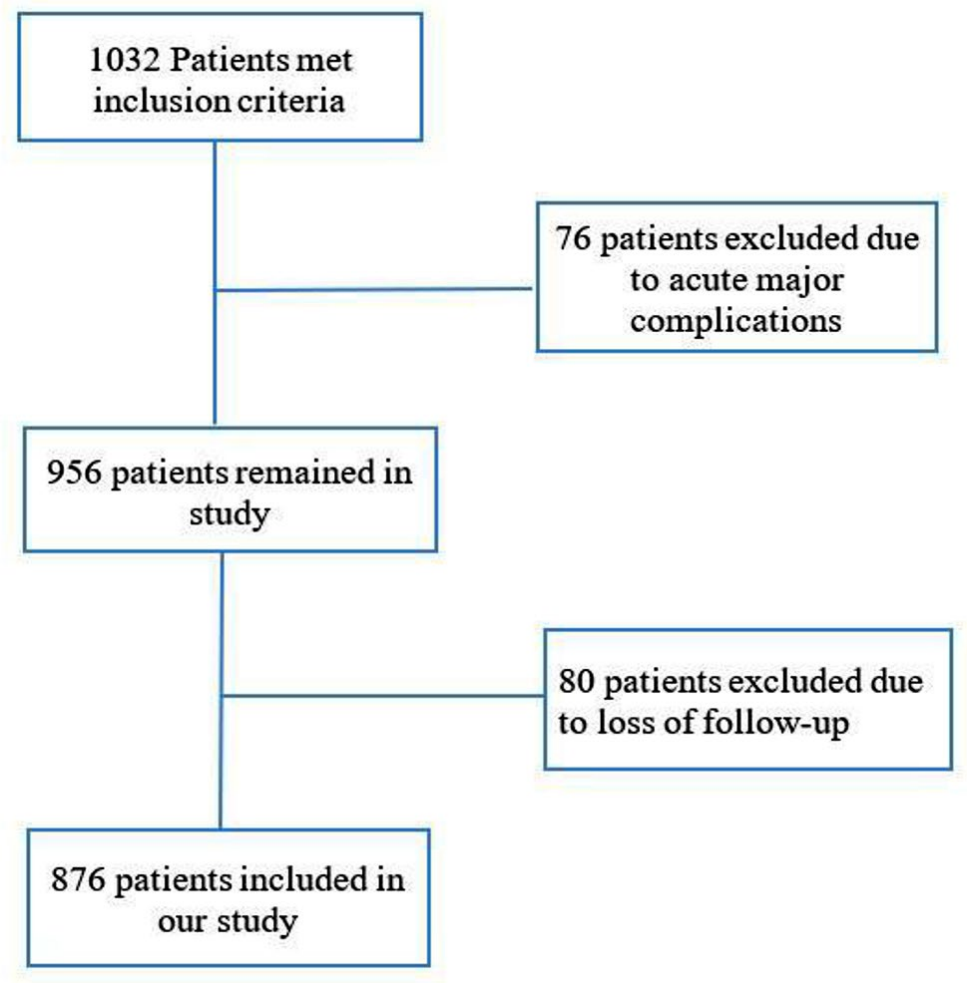
Retrospective Cohort study flowchart

**Fig. 2:**
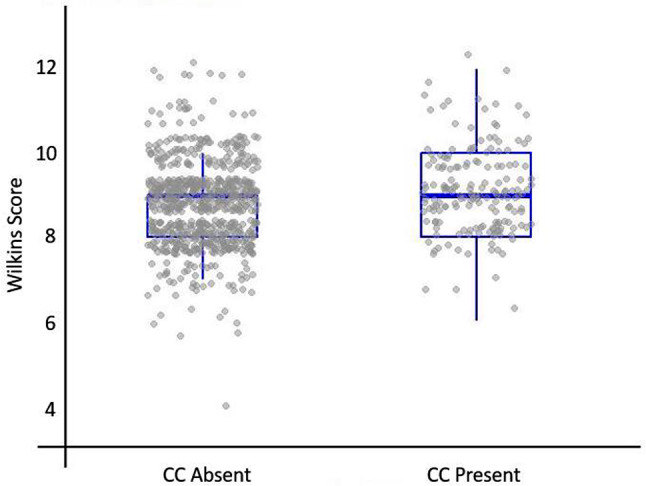
Wilkins score based on the presence or absence of CC. Box and whiskers represent the distribution of the Wilkins score in patients with and without CC. Dots represent the actual value of Wilkins score for each patient. CC: commissural calcification

The medical records of 1032 patients after PBMV were reviewed. Seventy-six patients were excluded due to acute major complications during the first 24 h after PBMV (32 patients developed tamponade, and 44 patients underwent surgical mitral valve replacement due to valve rupture and severe MR). Of the remaining 956 patients, 80 patients were excluded due to lack of follow-up. Finally, 876 patients were included in our analysis (Fig. 1). The median age of the patients was 46.0 [28–64] years, and 81.1% of the patients (*n* = 710) were females. Among participants, 333 patients (38.0%) had a Wilkins score of 8 or less were included in group I, 501 patients (57.2%) had a Wilkins score of 9 or 10 and were assigned to group II, and 42 patients (4.8%) with a score greater than 10 were classified as group III. Median Wilkins score was 9 [8.5–9.5] in the overall population. Additionally, 175 (20%) patients had CC prior to PMV, from which 95 (10.8%) were on the anterolateral commissure, 64 (7.3%) were on the posteromedial commissure, and the remaining 16 had bicommissural calcification on mitral valve leaflets. Baseline characteristics and echocardiographic measurements of patients based on Wilkins score and CC are summarized in Table 1. There was a significant difference in Wilkins score between patients with and without CC (*P* < 0.001) (Fig. 2).

**Table 1:** Baseline Characteristics and Echocardiographic Parameters of the Study

Variable	CC+	CC-	*P*-value	Wilkins group I	Wilkins group II	Wilkins group III	*P*-value
Age	46.70 ± 12.01	46.36 ± 12.39	0.74	45.08 ± 12.17	47.03 ± 12.30	49.83 ± 12.45	0.15
Female (%)	143(81.7%)	567 (82.1%%)	0.91	283(84.9%)	400(79.8%)	27(64.2%)	0.07
MVA	0.9 [0.75–1.05]	0.9 [0.75–1.05]	0.286	0.90 [0.75–1.05]	0.90 [0.75–1.05]	0.83 [0.69–0.98]	< 0.001
MVAI	0.41 [0.33–0.49]	0.41 [0.32–0.50]	0.079	0.42 [0.34-50]	0.41 [0.32–0.51]	0.34 [0.24–0.44]	0.003
sPAP	40 [32.5–47.5]	45 [35–55]	0.421	41 [33.5–48.5]	45 [36.5–53.5]	50 [37.5–62.5]	0.002
LVEF	55 [51–59]	55 [52.5–57.5]	0.050	55 [52.5–57.5]	55 [52.5–57.5]	50 [45–55]	0.309
Mean LAP	22 [16–28]	24 [18–30]	0.541	58.4 [52.9–63.9]	25 [19.5–30.5]	27.5 [22–33]	0.173

CC: Commissural Calcification; MVA: Mitral valve area; MVAI: Mitral valve area index; sPAP: Systolic pulmonary artery pressure; LVEF: Left ventricular ejection fraction; LAP: Left atrial pressure

At baseline, there was no significant difference in MVA, MVAI, PAP, mean LA pressure and LVEF between patients with and without CC (all *P* > 0.05). However, there was a significant difference in MVA, MVAI, and PAP among Wilkins score groups, with patients in Wilkins group III having lower MVA and MVAI and higher PAP compared to the other groups (*P* < 0.001, *P* = 0.003, and *P* = 0.002, respectively).

In the short-term and mid-term echocardiographic evaluation following the PBMV, MVA increased significantly compared with baseline measurements in total cohort (baseline: 0.9 [0.8-1.0]; short-term: 2.4 [2.2–2.6]; mid-term 2.1 [2.1–2.1]; both *p* < 0.0001).

### Mitral regurgitation at short-term follow up

**Fig. 3:**
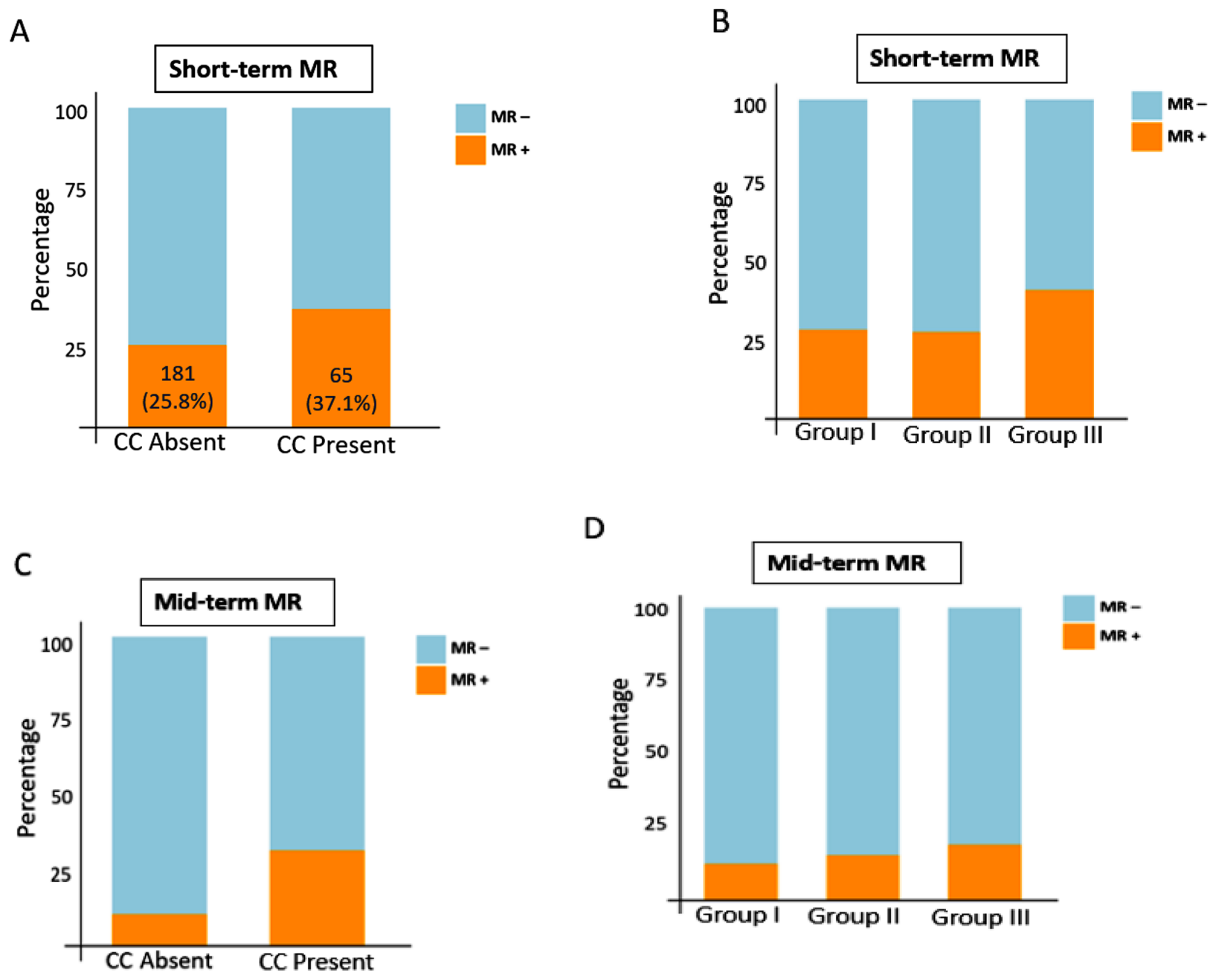
Figure 3 A-D Occurrence of short-term MR based on presence or absence of CC(**3 A**) and Wilkins Score (**3B**), and mid-term MR based on presence or absence of CC (**3 C**) and Wilkins Score (**3D**)

At the 6-month follow-up, 246 (28.1%) patients had symptomatic significant MR. The prevalence of symptomatic significant MR was 65 (37.1%) in patients with CC and 181 (25.8%) in those without CC (*P* = 0.003) in short-term follow up (Fig. 3A). Among Wilkins score groups, 93(27.9%) patients in group I, 136 (27.1%) patients in group II, and 17(40.5%) patients in group III developed symptomatic significant MR in short-term follow-up (*P* = 0.182) (Fig. 3B).

A univariable binary logistic regression revealed that patients with CC had significantly higher odds of experiencing short-term moderate and severe symptomatic MR that led to MVR (OR: 1.69, 95%CI 1.19–2.41, *P* = 0.003). This model explained 1.4% (Nagelkerke R^2^) of the variance in significant symptomatic MR incidence. In a multivariate logistic regression adjusted for age, sex, MVAI, PAP, and LVEF (X^2^ [6] = 49.11, *P* < 0.001), CC remained significantly associated with higher odds of short-term symptomatic significant MR (OR: 1.65, 95%CI: 1.15–2.37, *P* = 0.006). This model explained 7.4% (Nagelkerke R^2^) of the variance in MR at mid-term follow-up.

On the other hand, Wilkins groups II and III did not show significantly higher odds ratios for predicting short-term MR compared to group I (group II: OR: 0.96, 95%CI: 0.70–1.31, *P* = 0.784; group III: OR: 1.75, 95%CI: 0.90–3.39, *P* = 0.098).

### Mitral regurgitation at mid-term

At two years follow up, hundred and twenty-six (14.4%) patients had significant symptomatic MR that led to MVR. (Fig. 3C). There was a significant difference in the prevalence of MR in mid-term evaluation between patients with and without CC (CC: 54 (30.9%); no CC: 72 (10.3%), *P* < 0.001). Although there was an incremental increase in the prevalence of MR in mid-term follow up among Wilkins groups I through III, this difference was not statistically significant (group I: 41 (12.3%); group II: 77 (15.4%); group III: 8 (19%), *P* = 0.317) (Fig. 3D).

In univariable binary logistic regression analysis, Wilkins groups II and III had higher odds of developing MR in mid-term, however these differences were not statistically significant (group II: OR:1.29, 95%CI: 0.86–1.94, *P* = 0.216; and group III: OR: 1.68, 95%CI: 0.73–3.87, *P* = 0.227). In contrast, CC was significantly associated with higher odds of MR incidence at mid-term follow-up in univariate (OR: 3.90, 95%CI 2.61–5.83, *P* < 0.001), as well as multivariate model adjusted for age, sex, MVAI, PAP, and LVEF (X2 [6] = 49.11, OR: 3.78, 95%CI: 2.52–5.68, *P* < 0.001). Univariate and multivariate models could predict 8.2% and 9.7% (Nagelkerke R2) of the variation in MR occurrence at mid-term follow-up, respectively.

### Mitral valve restenosis at mid-term follow-up

**Fig. 4:**
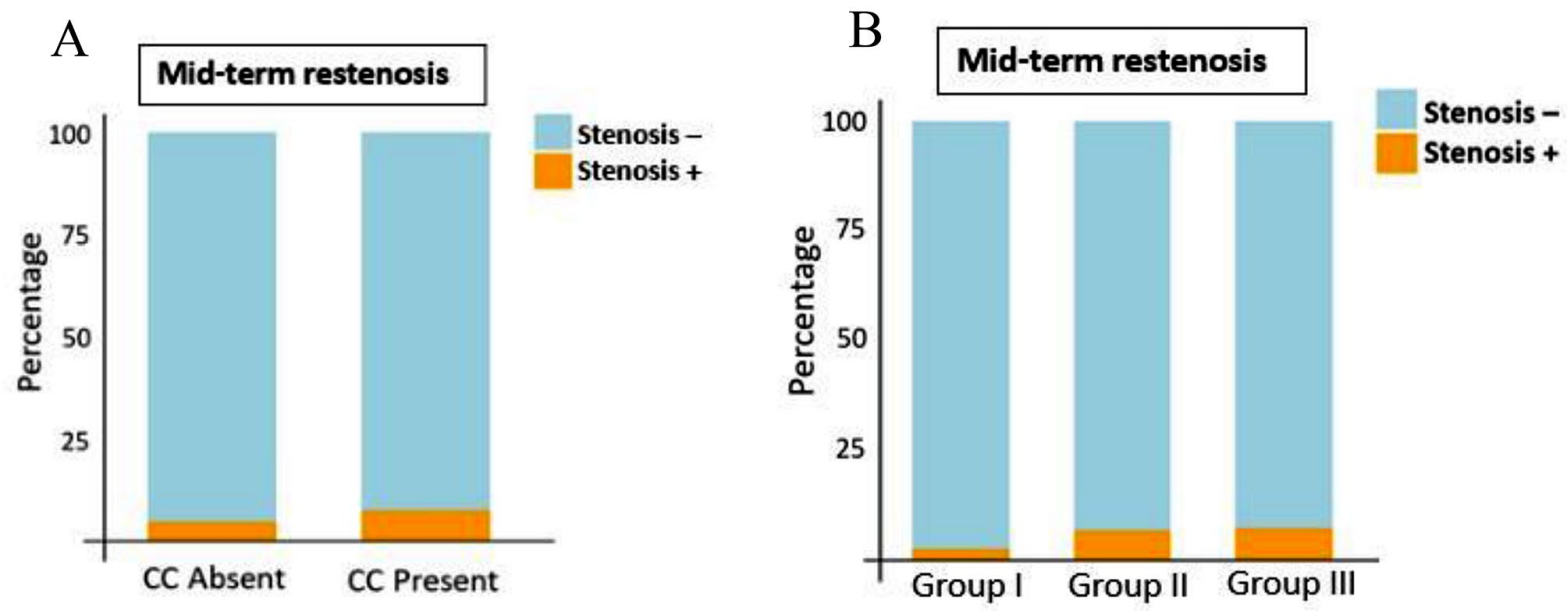
4**A** and 4**B** Occurrence of mitral valve restenosis based on presence or absence of CC (4A) and Wikins score (4B)

Mitral valve restenosis occurred in 45 (5.1%) patients during the 24-month follow-up. There was no significant difference in the prevalence of restenosis among patients with and without CC (CC: 13 (7.4%), no CC: 32 (4.6%), *P* = 0.125) (Fig. 4A). However, a significant difference in restenosis was observed among Wilkins score categories (*P* = 0.016). Patients in Wilkins group III had the highest occurrence rate of restenosis, followed by group II, and patients in group I had the lowest rate of restenosis (group III: 3 (7.1%), group II: 34 (6.8%), and group I: 8 (2.4%)) (Fig. 4B).

Binary logistic regression was used to examine the effects of Wilkins categories and CC on restenosis incidence at follow-up. While patients with CC showed higher odds of experiencing restenosis, this difference was not statistically significant (OR: 1.68, 95%CI: 0.861–3.27, *P* = 0.128).

The univariate model for Wilkins score was statistically significant (X^2^(df = 2) = 9.18; *P* < 0.001). In comparison to group I, the odds of experiencing restenosis in follow-up were significantly higher for group II (OR: 2.96,95%CI: 1.35–6.27, *P* = 0.007). However, the odds for group III, while elevated, did not reach statistical significance (OR: 3.13, 95%CI: 0.80-12.27; P: 0.103). In multivariable logistic regression model adjusted for age, sex, MVAI, PAP, and LVEF (X2 [7] = 14.52, *P* = 0.043), Wilkins group II still had significantly higher odds for developing restenosis (OR: 2.97, 95%CI: 1.35–6.55, *P* = 0.007). Patients in Wilkins group III had higher odds of having restenosis compared to group I, however this difference was not statistically significant (OR:3.49, 95%CI: 0.85–14.26, P:0.082).

## Discussion

### Short-term and mid-term follow up for MR severity

The main findings of our study are: 1. The importance of commissural calcification in predicting significant MR after PBMV in short-term and mid-term follow-up. 2. No statistically significant correlation between Wilkins score and significant MR following PBMV. 3. Mitral valve restenosis occurs more in patients with higher Wilkins score.

It has been shown that MR following PBMV is a common finding. However, significant MR following PBMV is a determinant of worse outcomes and need for mitral valve replacement. The precise mitral valve characteristics that can predict the outcomes remain to be elucidated [17–19].

Traditionally, the Wilkins score is used for pre-procedural risk assessment of patients undergoing PBMV [20]. However, the role of the Wilkins score in predicting clinical outcomes after PBMV is controversial. While several studies have shown Wilkins score to be a significant albeit relatively weak predictor of complications after PBMV [21–23], other studies have rejected any association between the mitral valve score and clinical outcomes and/or echocardiographic findings following PBMV [11, 24]. In our study, there was an incremental increase in the prevalence of significant MR in mid-term follow up of patients with higher Wilkins score, but it was not statistically significant which highlights that Wilkins score is not a strong predictor for occurrence of the significant MR following PBMV.

Previous studies have indicated that the optimal Wilkins score for PBMV is ≤ 8 [22]. Our study showed that in patients with a Wilkins score 9–10, PBMV can be performed with acceptable results if there is no CC. Furthermore, the semi-quantitative nature of the scoring system and lacking commissural assessment limits Wilkins score comprehensive predictive capacity. Although the Wilkins score does not directly assess the commissures, our study showed significantly higher scores in patients with CC. The degenerative disease of the commissures probably parallels with leaflet and subvalvular disease, with heavy generalized leaflet calcification likely involving the commissures.

In our study, presence of CC (unicommissural or bicommissural) was associated with a significantly higher rate of significant MR regardless of the total Wilkins score. Previous studies have confirmed that the mechanism underlying the increase in valve area during PMV involves the splitting of one or both fused mitral commissures, highlighting the need for examination of the CC as a separate entity with significant impact on PBMV outcomes [11, 12, 25].

In the study by Saturia et al., suggesting a new scoring system focused on the CC, they found that CC is associated with higher rates of MR after PBMV in patients with a Wilkins score of 8 or less [21]. Moreover, Fatkin et al. used TTE to demonstrate that commissural morphology resulted in a more accurate prediction of immediate outcomes compared to the Wilkins score in patients undergoing PBMV [11].

Notably, Anwar et al. used 3-Dimensional TTE for evaluating the structure of mitral valve to predict the outcomes of PBMV and reported that CC is related to a more severe MR in mid-term follow up, aligning with our findings [26]. Although we used 2-D TTE, potentially less precise than 3-D TTE, our results align with the study by Cannan et al., which reported that CC assessed by 2-dimensional echocardiography was a better predictor of significant MR compared to the Wilkins score. Their study also linked CC to higher rates of post-procedural complications, including moderate or severe MR, further supporting our observations [15].

While the Wilkins score remains a valuable screening tool for patients referred for PBMV, our results, in conjunction with prior studies, show the potential of CC as a reliable predictor of significant MR after PBMV and a possible indication for MVR.

### Mitral valve restenosis

Restenosis can develop after PBMV due to various factors such as disease progression, sub-optimal MVA after PBMV, and valve structure features like leaflets calcification and subvalvular calcification. However, mitral valve restenosis is not a common early complication following PBMV [27]. In a study by Sriram et al., restenosis rates were as low as 10% at 4 years, 18% at 5 years and 39% at the end of 7 years [28]. Similarly, in another study conducted by Wang et al., restenosis rate was 40% at the end of 6 years [29].

In our study, despite having a higher prevalence of restenosis, patients in Wilkins Group III did not have significantly higher odds of restenosis compared to Group I. However, patients in Group II had higher odds of restenosis than Group I. We speculate that the inconsistency in our results might be due to having fewer numbers of patients in Group III as well as short follow-up time after PBMV, resulting in low rates of restenosis at 24-months. Also, despite having higher rates of restenosis in patients with CC, CC was not a significant predictor of restenosis at 24-months. As we mentioned before this finding might be due to insufficient follow-up time after PBMV. Future studies with longer follow-up times are required to evaluate the predictiveness of CC on the incidence of restenosis after PBMV.

## Conclusion

In our study, commissural calcification was associated with higher rates of significant MR at short-term and mid-term follow-up after PBMV, while Wilkins score was not a strong predictive of MR. A detailed assessment of commissural calcification can provide beneficial prognostic information in patients undergoing PBMV in addition to the current valve scoring such as Wilkins score. It is reasonable to consider MVR when there is unfavorable MV morphology [19].

### Limitations

This study has a few limitations. First, this is a single-center retrospective study. However, the large number of cases with uniform approach in the center can compensate for some of the drawbacks. Second, the follow-up periods were relatively short, especially for the assessment of restenosis. Third, the CC was visually assessed however, this study was done by expert echocardiographers in a large referral center.

## Data Availability

All data used in this study is available and will be sent via email upon request. If the study data is required, you can contact the corresponding author.

## Abbreviations

PBMV: Percutaneous Balloon Mitral Valvuloplasty
CC: Commissural Calcification
MVA: Mitral Valve Area
MR: Mitral Regurgitation
MVR: Mitral Valve Replacement Surgery
LVEF: Left Ventricular Ejection Fraction
LVESD: Left Ventricular End-Systolic Diameter
LVEDD: Left Ventricular End-Diastolic Diameter
LA: Left Atrium
SPAP: Systolic Pulmonary Artery Pressure
